# Supplementary material for: Seroprevalence of SARS-CoV-2 Antibodies in Employees of Three Hospitals of a Secondary Care Hospital Network in Germany and an Associated Fire Brigade: Results of a Repeated Cross-Sectional Surveillance Study Over 1 Year
Source: Int J Environ Res Public Health. 2022 Feb 19;19(4):2402. doi: 10.3390/ijerph19042402 (PMC8878380; doi:10.3390/ijerph19042402)
Supplement: Supplementary file 1 [file ijerph-19-02402-s001.zip › ijerph-1551757-supplementary.pdf]

Supplementary Table S1: Age as risk factor for infection in the different observation periods.

a: June 2020 – September 2020

	SARS-CoV-2 antibodies			Statistics	
	**all**	**not detectable**	**detectable**	**OR**	**p.overall**
	n=1231	***n=1217***	***n=14***		
**Age:**					0.744
16-25	169 (13.7%)	167 (13.7%)	2 (14.3%)	Reference	
26-40	356 (28.9%)	351 (28.8%)	5 (35.7%)	1.19 [0.19;12.6]	
>40 years	706 (57.4%)	699 (57.4%)	7 (50.0%)	0.84 [0.16;8.32]	

b: October 2020 – January 2021

	SARS-CoV-2 antibodies			Statistics	
	**all**	**not detectable**	**detectable**	**OR**	**p.overall**
	n=1029	***n=893***	***n=136***		
**Age**					0.339
16-25	160 (15.5%)	136 (15.2%)	24 (17.6%)	Reference	
26-40	287 (27.9%)	244 (27.3%)	43 (31.6%)	1.00 [0.56;1.80]	
>40 years	582 (56.6%)	513 (57.4%)	69 (50.7%)	0.76 [0.45;1.32]	

c: February 2021 – June 2021

	SARS-CoV-2 antibodies			Statistics	
	**all**	**not detectable**	**detectable**	**OR**	**p.overall**
	n=714	***n=505***	***n=209***		
**Age**					0.052
16-25	110 (15.4%)	69 (13.7%)	41 (19.6%)	Reference	
26-40	204 (28.6%)	140 (27.7%)	64 (30.6%)	0.77 [0.46;1.29]	
>40 years	400 (56.0%)	296 (58.6%)	104 (49.8%)	0.59 [0.37;0.95]	

Note: date of birth is missing in 16 employees

Supplementary Table S2: Profession and institution as risk factor for infection.

Variable	SARS-CoV-2 antibodies							Statistics	
	**all**	**St. Vincenz Hospital Datteln**		**St. Laurentius Stift Waltrop**		**Vestische Kinder- und Jugendklinik Datteln**		**OR (95% CI)**	**p-value**
		not detected	detected	not detected	detected	not detected	detected		
	n=1699	n=725	n=133	n=260	n=59	n=487	n=35		
**profession**									
others*	297 (17.5%)	157 (21.7%)	18 (13.5%)	69 (26.5%)	13 (22.0%)	40 (8.21%)	0 (0.0%)	Reference	
nurse	906 (53.3%)	381 (52.6%)	77 (57.9%)	121 (46.5%)	25 (42.4%)	280 (57.5%)	22 (62.9%)	1.64 [1.09; 2.55]	0.021
MD	215 (12.7%)	79 (10.9%)	15 (11.3%)	29 (11.2%)	6 (10.2%)	79 (16.2%)	7 (20.0%)	1.64 [0.94; 2.85]	0.080
care worker	197 (11.6%)	93 (12.8%)	21 (15.8%)	16 (6.15%)	8 (13.6%)	55 (11.3%)	4 (11.4%)	2.07 [1.21; 3.54]	0.007
therapist	84 (4.9%)	15 (2.1%)	2 (1.5%)	25 (9.6%)	7 (11.9%)	33 (6.8%)	2 (5.7%)	1.55 [0.71; 3.21]	0.250

Note: * employees working in kitchen, administration, cleaning service and other professions with no contact to patients
